# Direct Posterior Bipolar Cervical Facet Radiofrequency Rhizotomy: A Simpler and Safer Approach to Denervate the Facet Capsule

**DOI:** 10.7759/cureus.2322

**Published:** 2018-03-14

**Authors:** Ovidiu Palea, Haroon M Andar, Ramon Lugo, Michelle Granville, Robert E Jacobson

**Affiliations:** 1 Anesthesiology and Pain Management, Provita Hospital; 2 Pain Management, Larkin Community Hospital; 3 Miami Neurosurgical Center, Coral Gables Surgical Center; 4 Miami Neurosurgical Center, University of Miami Hospital

**Keywords:** cervical radiofrequency rhizotomy, cervical facet joint, cervical spondylosis, facet capsule innervation

## Abstract

Radiofrequency cervical rhizotomy has been shown to be effective for the relief of chronic neck pain, whether it be due to soft tissue injury, cervical spondylosis, or post-cervical spine surgery. The target and technique have traditionally been taught using an oblique approach to the anterior lateral capsule of the cervical facet joint. The goal is to position the electrode at the proximal location of the recurrent branch after it leaves the exiting nerve root and loops back to the cervical facet joint. The standard oblique approach to the recurrent nerve requires the testing of both motor and sensory components to verify the correct position and ensure safety so as to not damage the slightly more anterior nerve root. Bilateral lesions require the repositioning of the patient's neck. Poorly positioned electrodes can also pass anteriorly and contact the nerve root or vertebral artery. The direct posterior approach presented allows electrode positioning over a broader expanse of the facet joint without risk to the nerve root or vertebral artery. Over a four-year period, direct posterior radiofrequency ablation was performed under fluoroscopic guidance at multiple levels without neuro-stimulation testing with zero procedural neurologic events even as high as the C2 spinal segment. The direct posterior approach allows either unipolar or bipolar lesioning at multiple levels. Making a radiofrequency lesion along the larger posterior area of the facet capsule is as effective as the traditional target point closer to the nerve root but technically easier, allowing bilateral access and safety. The article will review the anatomy and innervation of the cervical facet joint and capsule, showing the diffuse nerve supply extending into the capsule of the facet joint that is more extensive than the recurrent medial sensory branches that have been the focus of radiofrequency lesioning.

## Introduction

Background

Relieving pain in the cervical spine by performing cervical radiofrequency facet rhizotomy (RF), also called neurotomy, facet ablation, or facet denervation, is based on the finding that the recurrent sensory branch of the exiting nerve root has a "fixed" anatomic pathway that can be targeted to create radiofrequency lesions under fluoroscopic imaging [1-2]. The electrode targets the recurrent sensory branch in the anterior and ventral parts of the facet joint using oblique fluoroscopic imaging. Sensory and motor testing is used to both document that the correct segment is targeted and to ensure that the electrode is not affecting the exiting nerve root [2-3]. Clinical follow-up studies have shown that after relief from one or two temporary blocks with local anesthetic that localizes the affected spinal segment, the radiofrequency (RF) procedure relieves chronic neck pain. Common sources of pain that have responded to RF ablation include cervical disc degeneration, cervical and thoracic facet degeneration, soft tissue and facet injury from cervical whiplash, and post-laminectomy pain. In properly selected cases, pain relief has been consistently reported in 70% of the cases with relief lasting between seven and eight months [1-4]. If pain then recurs, after a successful initial procedure, a repeat procedure has a similar success rate [5]. Since the initial description of the RF target in the cervical spine, the basic technique and the target for the radiofrequency lesion have not changed [1-2]. There has been some improvement in electrode design, the type of current used to create the radiofrequency lesion, such as pulsed RF and cooled RF current, adding bipolar electrodes to make larger lesions along the facet capsule, as well as modifications to different-shaped electrode tips, but there has not been a change in the target [6-7].

Anatomy of the cervical spine, facet joint, and transitional zone between the cervical and thoracic spine

**Figure 1:**
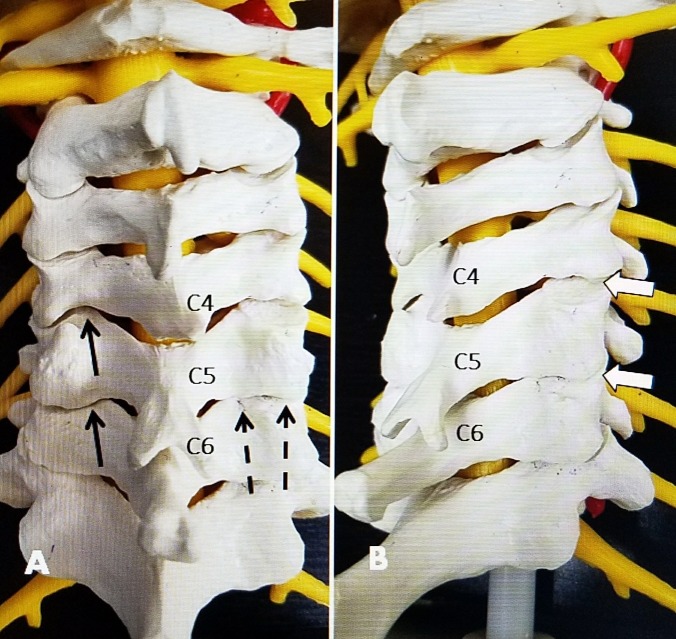
Anatomic model showing the approach angles for direct posterior radiofrequency versus the standard approach A: Posterior view of anatomic model showing inferior to the superior angle for two different electrode positions (single and double electrodes) along the posterior edge of the C4-5 and C5-6 facet joints. The two electrodes (dashed black arrows) are angled to match the inferior to superior inclination of the facet joint and positioned lateral to the interlaminar space. The two electrodes spaced between 4 mm and 8 mm apart create a larger bipolar lesion. On the opposite side, the solid black arrow shows a unilateral posterior approach at C4-5 and C5-6, angled again inferior to the superior and slightly medial to the lateral to create a lesion in the posterior edge of the capsule. B: Posterior-lateral oblique view similar to the approach for standard radiofrequency lesioning that targets the more anterior edge of the facet joint. This puts the electrode closer to the exiting nerve root.

Anatomic studies of the cervical spine and cervical facet joints demonstrate that the facet joint and capsule continue from the more ventral oblique and lateral side, forming a larger more horizontal posterior lip [8-9]. The cervical facet joint is inclined at a 30^o^ to 40^o^ inclination from posterior caudal to anterior cranial. However, the posterior part of the facet capsule is primarily horizontally oriented until C5-6. There is a slight change in orientation of the cervical facet from C5-6 to C6-7 as the facet joint becomes rotated slightly more laterally [10-11] (Figure 1).

**Figure 2:**
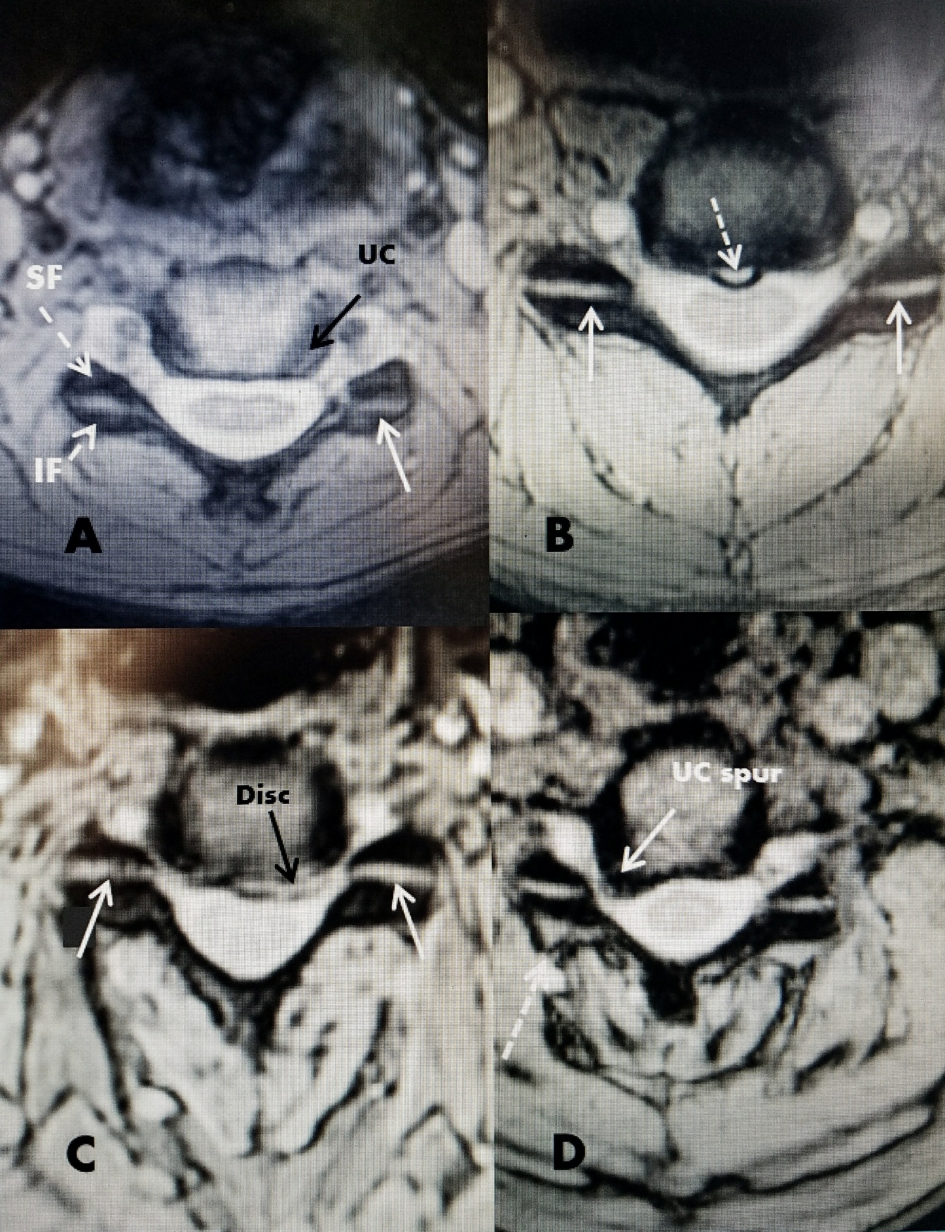
Axial magnetic resonance imaging (MRI) showing the relationship of the facet joint to other cervical structures A: Normal axial image of MRI cervical spine. The superior facet (SF) and inferior facet (IF) can be clearly seen. The uncovertebral joint (UC) can be seen lateral to the disc (solid black arrow). The uncovertebral joint emanates from the more lateral edge of the inferior vertebrae. The posteriorly located horizontal facet joint is seen (solid white arrow). B: Small midline annular tear (dotted white arrow). The facet joints are seen on both sides (solid white arrow). C: Herniated disc into the ventral floor of the cervical canal (solid black arrow). The facet joints are clearly seen (solid white arrow). D: Large posterior and lateral osteophyte from the back of the uncovertebral (UC) joint forming a spur into the intervertebral foramina (solid white arrow). The horizontal facet joint is clearly seen as in the other examples but there is minimal posterior enlargement of the facet capsule on the same side as the large osteophyte (dashed white arrow).

On magnetic resonance imaging (MRI) and computerized tomography (CT), the superior and inferior cervical facets from C2 to C6 can be seen to have a clear horizontal orientation on axial images. The shape and orientation remain unchanged regardless of the degree of segmental degenerative pathology. Biomechanically, the cervical facet joint provides mainly rotary and sagittal stability but minimal weight bearing [7-8]. This is markedly different from the lumbar facet joints that provide 20%-30% of the posterior column axial weight bearing in the normal spine but from 40%-60% of weight bearing with disc degeneration [7]. This shifting of spinal load on to the posteriorly located facet joints gradually leads to lumbar facet overgrowth and deformity. In contrast, in the cervical spine, disc degeneration is accompanied primarily by the overgrowth of the anterior and lateral uncovertebral joint without significant deformity of the facet joint [7,10] (Figure 2).

Neural supply to the facet joint and joint capsule

Anatomic studies originally made by Von Luschka in the 1850s noted the pathologic enlargement of the uncovertebral joint or von Luschka's joints in the cervical spine and the location of the recurrent nerve supplying the facet joint that passes posteriorly as the nerve root exits the neural foramina and then passes along the lateral vertebral body between the short pedicles, forming a multisegmental web posteriorly near the anterior edge of the cervical facet joint [1,12]. However, neurophysiologic studies and using detailed neural staining show that the recurrent branch is interconnected and part of a more diffuse neural network that extends over several spinal segments [12-13]. The primary posterior sensory ramus also receives a large sensory component from the posterior cervical multifidus muscles [13-14]. These fine nerve endings are mainly found in the posterior synovium and capsule of the facet joint, forming a rich neural network labeled Cruveilhier plexus and, in turn, connect with the meningeal nerves that supply sensory endings to the posterior and lateral disc annulus and spinal dura [15-16]. The recurrent sensory nerve also interconnects with the cervical sympathetic chain and the sino-vertebral nerve with multiple ascending and descending branches spreading over several spinal segments [14]. The sino-vertebral nerve re-enters the intervertebral foramen, innervating the annulus fibrosis of the intervertebral disc and the ligaments and periosteum of the spinal vertebra, carrying pain sensation from the annulus, the posterior longitudinal ligament, and the vertebral endplates [17-18]. Neural fibril staining shows the capsule of the facet joint, especially posteriorly, is richly innervated with nociceptive receptors [18]. Experimental and pathologic studies demonstrate that chronic facet degeneration and associated fibrous synovial capsule overgrowth is accompanied by inflammatory angiogenesis, which, in turn, leads to the development of marked neural fibril ingrowth. This nerve ingrowth and proliferation increase the abnormal nerve supply from the degenerated facet capsule [8,10,13,19]. Although ultimately, pain impulses pass through the recurrent sensory branches, they also can pass through other segments, including the sympathetic chain, meningeal nerve, and sino-vertebral nerve, creating a multisegmental sensory plexus [18-19]. Putting all these separate anatomic findings together indicates that the posterior facets and cervical spine are innervated through an extensive multi-innervated web. Targeting just a single branch of the recurrent sensory nerve alone may be insufficient to effectively denervate the multiple, other afferent pathways, which may account for the failures of radiofrequency rhizotomy [13,20]. To effectively "denervate" the facet capsule, not only one level above and below must be targeted, but it may be beneficial to add radiofrequency lesioning of the more horizontal posterior facet capsule [3,12]. As has been shown with pain from lumbar facet degeneration, targeting the facet capsule with its nerve-rich overgrown synovium may add additional benefits to the standard radiofrequency lesions [7].

## Technical report

In the process of performing over 200 diagnostic and therapeutic cervical facet blocks in patients seen in a neurosurgical practice, but using a more direct posterior approach to the posterior facet joint, it became apparent that the results posteriorly were similar to the more anterior oblique approach. Starting in 2012, cervical radiofrequency rhizotomy was performed using a direct posterior approach by placing the radiofrequency electrodes only along the posterior facet capsule rather than the more anterior oblique part of the facet joint. Initially, radiofrequency lesions were performed with one electrode, but, subsequently, it was found that there was space for using two electrodes to create bipolar lesions.

Procedural technique

**Figure 3:**
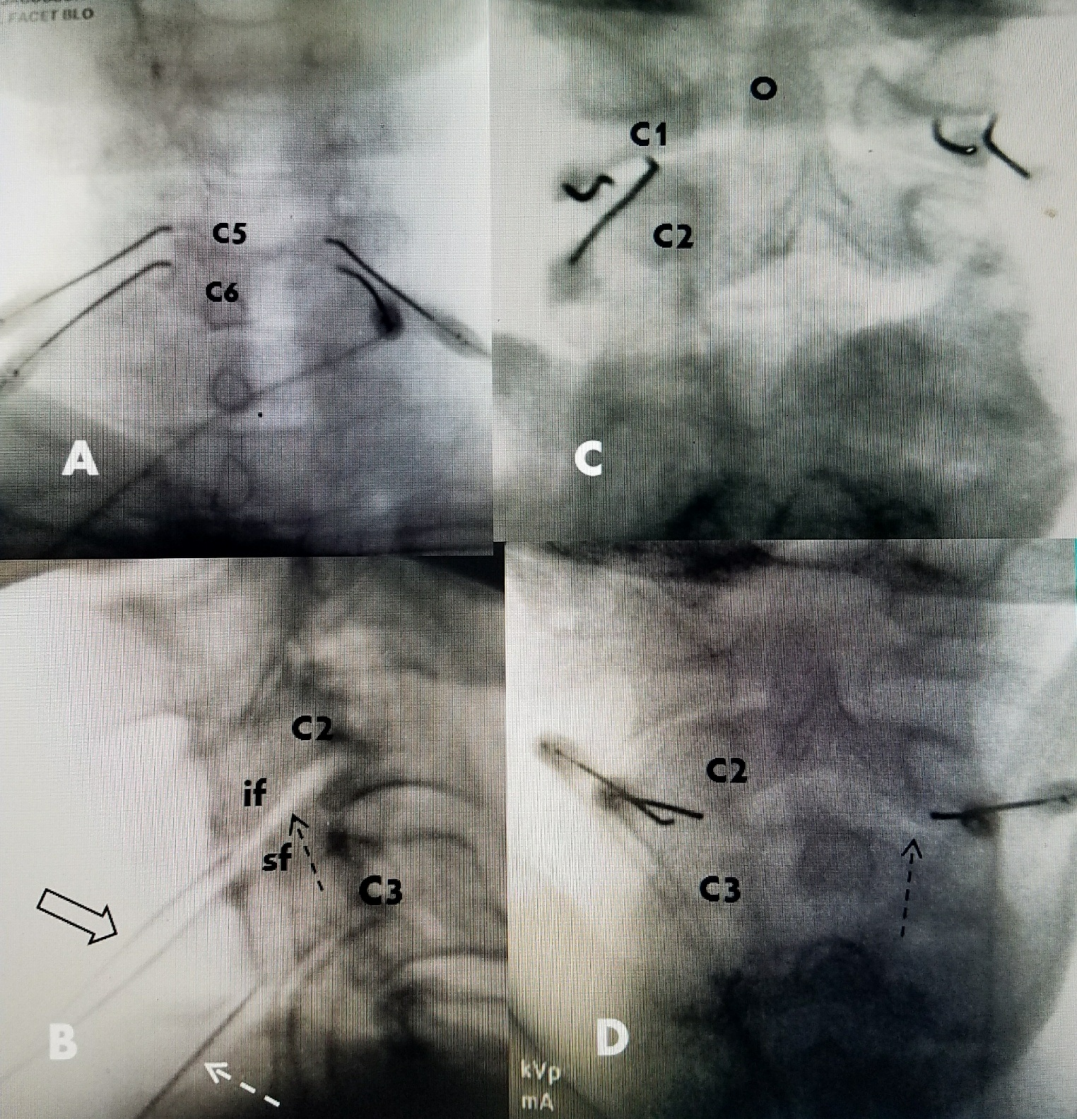
Intraoperative images of posterior electrode placement A: Patient having bipolar posterior medial facet radiofrequency lesions using two electrodes placed parallelly but vertically on the more oblique side of the C5-6 joint for bipolar lesioning. B: Lateral intraoperative film showing an angle (40 degrees) parallel to the direction of the joint from caudal to cranial to approach the posterior facet joint (dashed black arrow). The inferior facet (if) and superior facet (sf) are marked at C2-C3. The two bipolar electrodes are seen (wide black arrow) at C2-3 and the single unipolar electrode (dashed white arrow) can be seen at C3-C4, inclined at the same angle as the facet joint. C: Posterior bipolar C1-2 facet radiofrequency. Electrodes placed parallelly, about 4 mm apart, and horizontally, along the posterior facet capsule. The superior and more more anterior odontoid process (O) of C2 is clearly seen. D: Bipolar posterior facet radiofrequency electrodes at C2-3 for high cervical and occipital pain. The medial edge of the posterior facet capsule is seen (dashed black arrow).

All patients had at least one fluoroscopically guided facet block prior to undergoing facet radiofrequency rhizotomy. All blocks were performed using an approach that injected directly into the posterior cervical facet. The cervical spine was positioned under fluoroscopy with the neck straight and the forehead on a soft roll and slightly flexed position to open the facet joint. As confirmed by lateral imaging, even when patients had cervical kyphosis on standing radiographs or CT and MRI. In this position, the cervical spine often realigned into a straight line or more normal slight lordosis. It was also noted that all patients aligned in the anteroposterior (AP) plane. In fact, cervical scoliosis is very rare whereas it is very common in the lumbar spine, especially in patients with lumbar degeneration. For radiofrequency facet rhizotomy, an intravenous line was placed for conscious sedation, as needed by anesthesia. Next, under fluoroscopy by angling the tube from caudal to cranial, usually at between 20^o^ and 40^o^, depending on the spinal level, it was possible to visualize the posterior line of the facet joint. If needed, the inclination toward the facet capsule was confirmed using lateral fluoroscopy. The facet joint is always in line with the pedicle and at least 1 cm lateral to the midline spinous process. Based on this position and angulation, the skin was anesthetized with 1% lidocaine approximately 2 cm to 3 cm below the target level and then one or two Stryker (Kalamazoo, MI, USA), 100 to 150 cm, 10 mm curved tip radiofrequency electrodes were positioned in a parallel fashion horizontally over the posterior capsule of the facet joint. One electrode was placed just medial to the pedicle and the second, more lateral, electrode on the posterior and lateral margin of the posterior facet joint, with the two electrodes between 4 mm and 8 mm apart (Figure 1). With the electrode in contact with the bone, no sensory or motor testing was performed. Radiofrequency lesions were then made for 90 seconds at 80^o^ centigrade at each area. At the end of the procedure, 1 cc to 1.5 cc of 0.5% bupivacaine was injected into the radiofrequency site. Routinely, a minimum of three and a maximum of five levels underwent radiofrequency lesioning (Figure 3).

## Discussion

Over four years, a group of 18 patients underwent bipolar posterior cervical facet radiofrequency rhizotomy targeting the cervical posterior facet capsule without lesioning to the area of the recurrent sensory branch. No sensory or motor testing was performed at the time of the rhizotomy and no patient had neurologic complications. Four patients had previous anterior cervical fusions, one a posterior cervical laminectomy and laminoplasty, and the remaining had various degrees of multilevel degenerative cervical spondylosis with pain. All radiofrequency procedures were performed after preliminary posterior facet blocks using the same posterior approach as described in the technique section. A follow-up of this small group over four years showed 15/18, or 83%, never had another procedure performed and were classified by the patient visual analog scale (VAS) score as excellent or good results with residual pain between zero and three. Importantly, eight of the 18 were followed greater than two years and all but one patient had radiofrequency bipolar posterior capsule lesioning bilaterally at three or four levels. These observations suggest that performing radiofrequency procedures bilaterally, possibly using bipolar electrode placement, and at multiple levels is very effective. Only one patient in the group had residual pain complaints at long-term follow-up. This small series, but with long-term follow-up, in combination with the original use of the posterior capsular approach in over several hundred cervical facet blocks, demonstrates this is a safe technique and, more importantly, produces, at a minimum, similar results to the more anterior, oblique-angled approach. Performing radiofrequency ablation of the posterior facet capsule and using bipolar electrodes to create a larger lesion was previously shown to be effective for lumbar facet rhizotomy [7]. In the cervical radiofrequency rhizotomy group, there were no infections or hematomas and using this approach, without performing motor or sensory testing, there were no neurologic deficits from the procedure. The longest follow-up was between three to four years for seven patients.

This simple technique directly targets the larger and more easily accessible posterior part of the cervical facet joint [9,11]. Targeting the posterior facet capsule poses minimal risk to the more ventral exiting nerve root [12]. Creating a bipolar radiofrequency lesion in this area over several spinal segments is effective for targeting the diffuse neural plexus and refers sensation from the abnormal facet capsule and the posterior cervical muscles. Pathologic studies confirm that patients with abnormal facet joints also develop a marked ingrowth of fine sensory nerve endings in response to proliferative angiogenesis and fibro-proliferative overgrowth with inflammatory reaction [10]. The inflammatory pain and hypersensitivity caused by fine neural ingrowth can be denervated with radiofrequency lesions to the posterior facet capsule [7,10,17]. The direct positioning of the radiofrequency electrodes, especially when used in parallel, to create a larger bipolar lesion along the posterior facet capsule effectively and safely denervates this posterior region. This can be performed alone or in combination with the more anterior target to the recurrent sensory branch. In this limited group of patients, but with follow-up over four years, the effect of posterior capsule radiofrequency was as effective as the anterior oblique approach and there were minimal repeat procedures.

## Conclusions

Direct posterior cervical facet radiofrequency performed under fluoroscopic monitoring without preliminary neural sensory or motor testing is safe and easily allows the multisegmental and bilateral lesioning of three or four cervical spinal levels. Adding bipolar and multilevel lesioning to the posterior facet capsule was effective and safe in providing long-term pain relief in a small patient group.This technical study describes the steps of this approach and the fact that there was no evidence of neurologic risk. Patient follow-up demonstrated similar results as those reported by the oblique technique. These patients were selected for the procedure and followed within a neurosurgical practice rather than through a pain management clinic. A smaller subgroup of these patients, followed between two and four years after the multilevel and bilateral posterior cervical capsule radiofrequency rhizotomy, demonstrated that the pain relief was maintained.
